# Effect on cardiac function among patients with type 2 diabetes following high-dose mineralocorticoid receptor antagonist using echocardiography; data from the MIRAD randomized clinical trial

**DOI:** 10.1186/s12872-023-03183-1

**Published:** 2023-03-31

**Authors:** Niels H. Brandt-Jacobsen, Marie Louise Johansen, Jon J. Rasmussen, Morten Dalsgaard, Thomas Kumler, Jens Faber, Patrick Rossignol, Morten Schou, Caroline Kistorp

**Affiliations:** 1 Department of Endocrinology, Copenhagen University Hospital - Rigshospitalet, Copenhagen, Denmark; 2 Department of Clinical Medicine, Faculty of Health and Medical Sciences, University of Copenhagen, Copenhagen, Denmark; 3 Department of Endocrinology-Internal Medicine, Copenhagen University Hospital - Herlev and Gentofte Hospital, Herlev, Denmark; 4 Department of Cardiology, Copenhagen University Hospital - Herlev and Gentofte Hospital, Herlev, Denmark; 5 Université de Lorraine, Nancy, France; 6 Department de Défaillance Cardiovasculaire Aiguë et Chronique, L’Institut National de la Santé et de la Recherche Médicale (URM-S 116), Nancy, France; 7 Centre Hospitalier Régional Universitaire, Nancy, France; 8 French Clinical Research Infrastructure Network Investigation Network Initiative - Cardiovascular and Renal Clinical Trialists, Nancy, France; 9 Centre d’Investigation Clinique Plurithématique 1433, L’Institut National de la Santé et de la Recherche Médicale, Nancy, France

**Keywords:** Mineralocorticoid receptor antagonist, High-dose eplerenone, Global longitudinal strain, Systolic and diastolic function

## Abstract

**Background:**

Early heart failure prevention is central in patients with type 2 diabetes, and mineralocorticoid receptor antagonists (MRAs) have shown to improve prognosis. We investigated the effect of high-dose MRA, eplerenone, on cardiac function and structure in patients with type 2 diabetes and established or increased risk of cardiovascular disease but without heart failure.

**Methods:**

In the current randomized, placebo-controlled clinical trial, 140 patients with high-risk type 2 diabetes were randomized to high-dose eplerenone (100–200 mg daily) or placebo as add-on to standard care for 26 weeks. Left ventricular systolic and diastolic function, indexed left ventricular mass (LVMi), and global longitudinal strain (GLS) were assessed using echocardiography at baseline and after 26 weeks of treatment.

**Results:**

Of the included patients, 138 (99%) had an echocardiography performed at least once. Baseline early diastolic in-flow velocity (E-wave) indexed by mitral annulus velocity (e’) was mean (SD) 11.1 (0.5), with 31% of patients reaching above 12. No effect of treatment on diastolic function was observed measured by E/e’ (0.0, 95%CI [-1.2 to 1.2], *P* = 0.992) or E/A (-0.1, 95%CI [-0.2 to 0.0], *P* = 0.191). Mean left ventricular ejection fraction (LVEF) at baseline was 59.0% (8.0). No improvement in systolic function was observed when comparing groups after 26 weeks (LVEF: 0.9, 95%CI [-1.1 to 2.8], *P* = 0.382; GLS: -0.4%, 95%CI [-1.5 to 0.6], *P* = 0.422), nor in LVMi (-3.8 g/m^2^ 95%CI [-10.2 to 2.7], *P* = 0.246).

**Conclusion:**

In the present echo sub-study, no change in left ventricular function was observed following high-dose MRA therapy in patients with type 2 diabetes when evaluated by conventional echocardiography.

**Trial registration:**

Date of registration 25/08/2015 (EudraCT number: 2015–002,519-14).

**Supplementary Information:**

The online version contains supplementary material available at 10.1186/s12872-023-03183-1.

## Background

In patients with type 2 diabetes, the risk of heart failure hospitalization as the first cardiovascular event exceeds myocardial infarction by approximately a factor four [1]. Undiagnosed cardiovascular disease, especially heart failure, in type 2 diabetes is thus, an established risk, which results in severely impaired prognosis. Changes in cardiac structure and diastolic dysfunction precedes heart failure with preserved ejection fraction (HFpEF), being one of the hallmarks of the first stages in its classification and one of the earliest markers of left ventricular dysfunction [2, 3]. While evidence-based preventive treatment options are limited [4–6], treatment with renin-angiotensin-system (RAS) inhibitors (i.e., angiotensin converting enzyme-inhibitors, angiotensin receptor blockers) and sodium-glucose cotransporter 2-inhibitors are now recommended for high-risk type 2 diabetes patients by current guidelines as early prevention and mitigating the cardiac risk [2, 7]. In contrast, mineralocorticoid receptor antagonists (MRAs) has long remained as a fourth-in-line antihypertensive in the absence of overt heart failure with reduced ejection fraction (HFrEF) [2, 7].

Experimental evidence has proven that inhibition of the mineralocorticoid receptor is an important therapeutic target in the prevention of cardiovascular disease in animal models of diabetes, obesity and early heart failure [8–11]. Further, a large body of evidence from clinical studies have established that MRA improves prognosis in patients with documented HFrEF [12–14], while the available evidence on use prior to developing HFrEF is less clear [6]. The use of MRAs has been suggested to improve cardiac function in patients across varying stages of heart failure using echocardiography [15, 16]. Interestingly, it has recently been suggested that the beneficial effects of MRAs may be further amplified with concurrent metabolic disease [17], with increased clinical benefits in patients with obesity, or type 2 diabetes [18]. While previous trials have showed increased effects of higher doses of an MRA [19, 20], a clear benefit may depend on the presence of concurrent metabolic dysfunction. Therefore, accruing further knowledge on the early effects of MRAs on the adverse cardiac changes in patients with type 2 diabetes is crucial to understand the possible clinical impact of MRAs in the prevention of heart failure.

In this substudy of the Mineralocorticoid Receptor Antagonist in type 2 Diabetes (MIRAD) trial, we aimed to investigate whether high-dose eplerenone as an add-on therapy improves left ventricular function evaluated by echocardiography. In the present study we report the prespecified, secondary endpoints of left ventricular systolic and diastolic function, including global longitudinal strain (GLS), after 26 weeks of treatment with the MRA, eplerenone.

## Methods

### Trial design

The presented results are based on data from the MIRAD trial, a double-blinded, placebo-controlled, randomized clinical trial. Its design has previously been described in detail while reporting the primary outcome liver fat content [21]. Briefly, the study enrolled 140 patients with type 2 diabetes and a high risk of or established cardiovascular disease from an outpatient clinic at a university hospital in Copenhagen, Denmark. Patients were randomized using a 1:1 block-randomization (block-size: 10), created by a third party, Glostrup Pharmacy. Patients were randomized to maximally-achievable blockade of the mineralocorticoid receptor with the MRA, eplerenone (100–200 mg daily), or dose-matched placebo for 26 weeks by primary subinvestigator, MLJ, blinded to treatment. The study intervention followed a fixed-triation paradigm from an initial dose of 50 mg, and dose increases every 2 weeks of 50 mg until maximal tolerable dose was achieved. As previously reported in detail [22], patient safety and compliance was closely monitored with measurements of plasma electrolytes and estimated glomerular filtration rate at each step of titration until the trials 10^th^ week, and thereupon every four weeks until completion of the trial as a part of the prespecified protocol. The treatment drug and placebo, provided by Glostrup Pharmacy, could not be distinguished from each other. The trial included patients with a diagnosis of type 2 diabetes and concurrent cardiovascular disease or a high risk thereof defined by the following key inclusion criteria: previous myocardial infarction, significant stenosis on coronary angiography, previous stroke or transient ischemic attack at least 3 months prior to randomization, presence of peripheral artery disease, NT-proBNP ≥ 70 ng/L [23], or albuminuria defined as urinary albumin–creatinine ratio ≥ 30 mg/g. Key exclusion criteria were HFrEF (left ventricular ejection fraction [LVEF] < 40%), potassium ≥ 5.0 mmol/L, or impaired kidney function (estimated glomerular filtration rate of ≤ 40 mL/min/1.73 m^2^).

### Acquisition and processing of cardiac images

Transthoracic echocardiography was performed by experienced cardiologists using a Vived E9 appropriated to the current study (MS, MD, TK) (GE Vingmed Ultrasound, Norway) in accordance with current guidelines [24]. Two-dimensional images were acquired in three apical views (2, 4-chamber and long-axis) and two parasternal views (long and short axis). Left ventricular end-diastolic (EDV), end-systolic (ESV), stroke volume (SV), LVEF, and maximal left atrial volume were derived using the modified Simpson’s method. Left ventricular diastolic function was assessed using pulsed-wave Doppler to measure E-wave, E-wave deceleration time, and late atrial filling (A-wave). The e’ used in analysis was derived using the mean of measurements at the base of interventricular septum and from the base of the lateral wall measured in the 4-chamber view using tissue Doppler. GLS was derived from all three apical views using speckle tracking. Radial strain and circumferential strain analysis were not performed. All image post-processing was performed offline by a single investigator (NBJ) masked from treatment-group allocation on commercially available software (Echopac BT 12.1.0; GE, Norway). Post-processing measurements were performed three times at each visit and averaged with the exception of GLS, which was performed once. Left ventricular mass (LVMi) and left atrial volume were indexed to body surface-area using the formula by Devereux et al. [25], with hypertrophy defined as above 115 g/m^2^ for men and 95 g/m^2^ [24] and an enlarged left atrium defined as above 34 mL/m^2^. Additional echocardiographic measures of left ventricular and right ventricular function, including right ventricular free wall GLS, are reported as part of the performed echocardiography (Baseline characteristics according to treatment group: Supplementary table 1; changes from baseline: Supplementary table 2).

### Study outcomes

We report the predefined, secondary endpoints of the MIRAD trial: change in left ventricular function by echocardiography (LVEF, EDV, ESV, e’, GLS), with additional focus on diastolic function (E/e’, E/A, left atrial volume) and LVMi.

### Statistical analyses

We used a constrained linear mixed model to evaluate treatment effect (or between-group difference) on all echocardiographic parameters. To account for correlation between repeated measurements on the same subject, an unstructured covariance pattern was assumed. Analyses were performed as intention-to-treat. Missing values were deemed to be missing at random and were implicitly imputed by maximum likelihood-estimation in the linear mixed model. Sensitivity analyses using the complete-case cohort were performed, but did not change the conclusions based on the primary analyses (Supplementary table 3). Potential effect modification of concurrent medication at baseline (RAS-inhibitors or beta-blockade) was evaluated by performing additional analyses on selected outcomes (Left atrial volume indexed to body surface area, E/e’, LVMi), however, no significant interaction was found (Supplementary table 4).

Descriptive characteristics are reported by mean and standard deviation (SD) unless otherwise specified. Baseline characteristics were tested for baseline-imbalance using student’s t-test for continuous or log-transformed variables and fisher’s exact test for counts. Changes from baseline and treatment effects are preferably reported as the mean, a 95% confidence interval [95%CI] and two-tailed unadjusted p-values. A p-value below 0.05 considered as statistically significant. Data-transformation was used to adhere to model assumptions and results are presented after de-transformation. Statistical analyses and graphical design were performed using SAS (version 9.4 TS 1M5) (SAS Institute Inc., Cary, NC, USA), GraphPad Prism 8, version 8.1.0 (GraphPad Software, San Diego, CA, USA), and www.biorender.com (Toronto, Ontario, Canada).

## Results

### Participant characteristics

A total of 140 patients were randomized to eplerenone or placebo between October 2015 and November 2017. At baseline, an echocardiography was performed on 138 patients (99%), missing two patients from the eplerenone group. Of these patients, an echocardiography was performed on 124 patients after week 26 (89%) (eplerenone *n* = 61; placebo *n* = 63). In the final analysis, 138 patients with at least one echocardiography were included (Supplementary Fig. 1: Flowchart).

As summarized in Table 1, the patients included in the analyses were well-balanced at baseline according to treatment groups. Almost half (44%) of the included patients had established cardiovascular disease at baseline, with the majority having coronary heart disease (28%). Of the patients included 78% received RAS inhibitors, with 36% and 37% receiving Ca-antagonists or Beta-blockers, respectively. Prior to enrollment, patients were required to receive a stable treatment regimen in accordance with anti-diabetic standard of care, and at baseline patients were fairly well-regulated with a mean (SD) HbA1c at baseline of 59.5 (14.5). Metformin was the most prevalent therapy (82%), followed by insulin (51%), glucagon-like peptide 1-receptor agonist (32%), SGLT2-i (23%), while patients prescribed dipeptidyl peptidase IV-inhibitors or sulfonylureas were less common (Table 1).

**Table 1:** Baseline characteristics according to treatment group

	**Placebo**		**Eplerenone**	
**Demographics**	70		68	
Age, years	64.1	(8.7)	62.8	(10.0)
Sex, Male (%)	50	(71)	52	(76)
BMI, kg/m^2^	30.8	(4.6)	30.5	(3.9)
BSA, m^2^	2.1	(0.2)	2.1	(0.2)
Duration of diabetes, years^a^	11	(5 to 16)	12	(7 to 16)
HbA1c, mmol/mol^**b**^	57.2	[53.9 to 60.6]	58.6	[55.4 to 61.9]
eGFR, mL/min/1.73m^2^	83.4	(16.6)	86.9	(20.6)
24 h-blood pressure, mmHg	129/77	(12.9/10.1)	129/78	(12.8/8.5)
Cardiovascular disease, n (%)	33	(47%)	31	(46%)
Chronic heart disease^c^	21	(30%)	19	(28%)
Stroke	4	(6%)	8	(12%)
Periferal arterial disease	11	(16%)	5	(7%)
UACR, mg/g^**b**^	19.2	[13.3 to 27.7]	17.2	[12.0 to 24.5]
NT-proBNP, ng/L^**b**^	83.7	[66.0 to 106.2]	69.5	[55.6 to 86.9]
**Echocardiography**				
LVEF, %	58.3	(8.5)	59.2	(7.4)
GLS, %	-15.5	(3.7)	-15.1	(3.1)
LVM, g	194.4	(43.9)	195.7	(54.9)
LVMi, g/m^2^	93.9	(19.7)	92.6	(25.7)
LVH, n (%)	12	(17)	10	(15)
E/e'	11.3	(4.9)	10.9	(3.9)
E/e’ > 12, n (%)	22	(33)	20	(29)
e', cm/s	6.9	(1.6)	7.4	(1.7)
LA volume, mL	53.0	(19.4)	53.2	(16.6)
LAi, < 34 mL/m2, n (%)	8	(12)	7	(10)
**Medication, *n* (%)**				
RAS-inhibitors	58	(83%)	52	(76%)
Calcium-antagonist	29	(41%)	21	(31%)
Beta blockers	25	(36%)	24	(35%)
Diuretics	38	(54%)	33	(49%)
Metformin	56	(80%)	58	(85%)
GLP1-RA	25	(36%)	19	(28%)
SGLT2-inhibitors	9	(13%)	13	(19%)
Insulin	37	(53%)	33	(49%)
Sulfonylarea	13	(19%)	17	(25%)
Dipeptidyl peptidase-IV inhibitors	6	(9%)	10	(15%)
Cholesterol lowering	63	(90%)	63	(93%)
Potasium-lowering therapy^d^	0	(0%)	0	(0%)

Values are reported as mean (SD) unless otherwise specified. ^a^Median (interquartile range). ^b^Geometric mean [95%CI] ^c^Established chronic heart disease was defined as previous cardiovascular/coronary stenosis requiring intervention (e.g. PCI or CABG) or previous myocardial infarction. ^d^Therapy specifically lowering potassium levels were not considered during the conduct of the trial

Abbreviations: *BMI* Body mass index, *BSA* Body surface area, *eGFR* Estimated glomerular filtration rate, *UACR* Urine-albumin-creatinine-ratio, *NT-proBNP* N-terminal proBrain Natiuretic Peptide, *LVEF* Left ventricular ejection fraction, *GLS* Global longitudinal strain, *LVM* Left ventricular mass, *LVMi* Left ventricular mass indexed to surface area, *LVH* Left ventricular hypertrophy, *E/e’* Early diastolic inflow velocity indexed to mitral annulus velocity, *e’* Mitral annulus velocity, *LA volume* Left atrial volume, *LAi* Left atrium indexed to body surface area, *RAS* Renin-angiotensin-system, *GLP1-RA* Glucagon-Like-Peptide 1-receptor agonist, *SGLT2* Sodium-glucose cotransporter-2

As previously reported, the fixed titration regimen was well-tolerated. Among patients with an estimated glomerular filtration rate above 60 mL/min/1.73m^2^, the vast majority (91%) received a dose of 100 mg daily or above from week 8 until week 26. In comparison, among patients with an estimated glomerular filtration rate below 60 mL/min/1.73m^2^ (*n* = 12), only 29% in the eplerenone group received 100 mg at week 26 [22]. No patients experienced a serious adverse event deemed to be caused by active treatment during the conduction of the trial, and no patient needed investigator-initiated termination of treatment due to hyperkalemia or a decrease in estimate glomerular filtration rate.

### Diastolic function

**Fig. 1:**
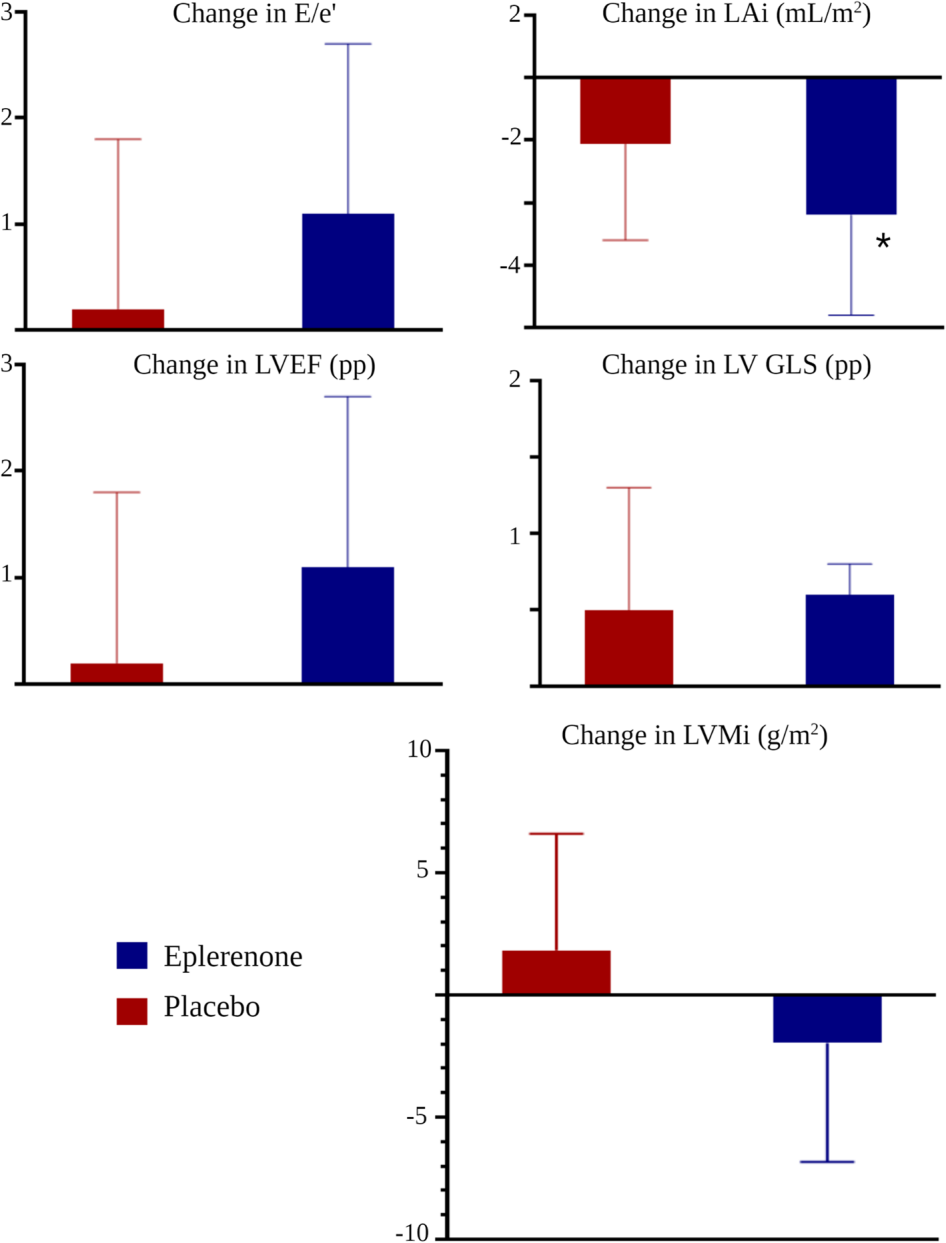
Change from baseline according to treatment group (Panel A-E). Values shown as mean with corresponding upper/lower 95%CI-limit using a constrained linear mixed model. Patients' E/e' (A) did not change in either group in E/e’ (**A**), however, a significant change was observed in left atrial volume (**B**), though only in the eplerenone group when compared to baseline. There was no overall improvement in diastolic function. Furthermore, no improvement was found in systolic function using LVEF (**C**) or GLS (**D**) (shown in percentage points) nor was an overall treatment effect found in LVMi when comparing groups after 26 weeks (**E**). * Significant decrease from baseline

At baseline, mean (SD) E/e’ was 11.1 (0.5), and patients with diastolic dysfunction, defined by an E/e’ above 12, comprised 31% of the study population (Table 1). The early and late maximal diastolic ventricular in-flow, peak E-wave and peak A-wave, did not change when compared to baseline within the eplerenone group or as an effect of treatment. Similarly, no change was observed in maximal velocity of the mitral annulus, e’, within groups or as an effect of treatment. Consequently, no treatment effect of E/e’ or E/A was observed when comparing groups at week 26 (E/e’: 0.0, 95%CI [-1.2 to 1.2], *P* = 0.992; E/A: -0.1, 95%CI [-0.2 to 0.0], *P* = 0.191) (Table 2, Fig. 1). Mean left atrial volume at baseline was 53.1 mL (18.0), with 11% presenting with increased indexed atrial volume (Table 1). No change was observed in left atrial volume from baseline, with an effect of treatment when comparing groups at week 26 of -2.3 mL (95%CI [-6.6 to 1.9], *P* = 0.277) (Table 2, Fig. 1).

**Table 2:** Changes in cardiac function from baseline at week 26 and effect of treatment

	**Baseline**			**Placebo**			**P**	**Eplerenone**			**P**	**Treatment effect**			**P**
**Diastolic function**															
E-wave, m/s	0.8	[0.7 to	0.8]	0.0	[0.0 to	0.0]	0.530	0.0	[0.0 to	0.0]	0.855	0.0	[0.0 to	0.1]	0.752
A-wave, m/s	0.8	[0.7 to	0.8]	0.0	[0.0 to	0.0]	0.448	0.0	[0.0 to	0.1]	0.180	0.0	[0.0 to	0.1]	0.131
e'-wave, cm/s	7.1	[6.8 to	7.4]	0.0	[-0.3 to	0.4]	0.944	0.1	[-0.2 to	0.5]	0.521	0.1	[-0.4 to	0.6]	0.669
E/e’-ratio	11.2	[10.4 to	11.9]	0.2	[-1.4 to	1.8]	0.802	1.1	[-0.5 to	2.7]	0.191	0.0	[-1.2 to	1.2]	0.992
E/A-ratio	1.1	[1.0 to	1.2]	0.0	[-0.1 to	0.1]	0.682	-0.1	[-0.1 to	0.0]	0.162	-0.1	[-0.2 to	0.0]	0.191
Deceleration, ms	264.0	[251.6 to	276.4]	2.8	[-13.8 to	19.3]	0.742	14.4	[-2.3 to	31.1]	0.089	11.7	[-9.7 to	33.1]	0.282
Atrial volume, mL	53.2	[50.1 to	56.2]	-2.1	[-5.2 to	1.0]	0.187	-4.4	[-7.6 to	-1.3]	0.006	-2.3	[-6.6 to	1.9]	0.277
**Systolic function**															
EF, %^a^	58.7	[57.4 to	60.1]	0.2	[-1.4 to	1.8]	0.802	1.1	[-0.5 to	2.7]	0.191	0.9	[-1.1 to	2.8]	0.382
EDV, mL	87.5	[83.6 to	91.4]	1.9	[-2.3 to	6.0]	0.379	-2.1	[-6.2 to	2.1]	0.319	-4.0	[-9.6 to	1.7]	0.166
ESV, mL	36.6	[34.4 to	38.9]	0.1	[-2.2 to	2.4]	0.938	-1.9	[-4.2 to	0.4]	0.113	-2.0	[-5.0 to	1.1]	0.201
GLS, %^a^	-15.3	[-15.8 to	-14.7]	0.5	[-0.3 to	1.3]	0.220	0.06	[-0.7 to	0.8]	0.876	-0.4	[-1.5 to	0.6]	0.422
**Cardiac structure**															
LVMi, g/m^2^	92.9	[89.1 to	96.8]	1.8	[-2.9 to	6.6]	0.449	-2.0	[-6.8 to	2.9]	0.426	-3.8	[-10.2 to	2.7]	0.246

Values reported are mean with corresponding 95%CI using a constrained linear mixed model with between-group differences noted as treatment effects ^a^Change reported in percent points

*Abbreviations*: E-wave, early diastolic inflow velocity; A-wave, late diastolic inflow velocity; e’, mitral annulus velocity; *GLS *Global longitudinal strain, *EF *Ejection fraction, *EDV *End-diastolic volume, *ESV *End-systolic volume, *LVMi *Left ventricular mass indexed to body surface area

### Systolic function and left ventricular mass

At baseline, mean LVEF was 59.0% (8.0), with 86% of the population having an LVEF above 50%. Following the addition of eplerenone, neither EDV nor ESV changed from baseline in either group or when comparing groups at week 26. There was no change in LVEF when comparing groups at week 26 (0.9, 95%CI [-1.1 to 2.8], *P* = 0.382) (Table 2, Fig. 1). Baseline mean GLS (SD) was -15.3% (3.4), and no improvement in the eplerenone group was observed when compared with baseline (0.06, 95%CI [-0.7 to 0.8], *P* = 0.876). As an effect, no treatment effect was observed on GLS when comparing groups at week 26 (-0.4, 95%CI [-1.5 to 0.6], *P* = 0.422, Fig. 1). Patients with left ventricular hypertrophy at baseline comprised 16% of the total cohort, and the LVMi population-mean at baseline was 92.8 g/m^2^ (22.7). The addition of eplerenone did not elicit an observable change in LVMi when comparing groups at week 26, with a non-significant treatment effect of -3.8 g/m^2^ (95%CI [-10.2 to 2.7], *P* = 0.246) (Table 2, Fig. 1).

Evaluating additional measures of left ventricular function obtained using a conventional echocardiography protocol, we observed no significant effect of treatment when comparing groups at 26 weeks (Supplementary table 2). Although, we did observe a significant treatment effect in right ventricular GLS, no significant treatment effects were observed in the additional measures of right ventricular function to corroborate this finding (right ventricular S’, TAPSE, TR) (Supplementary table 2).

## Discussion

In the present echocardiography sub-study of the MIRAD trial, no improvement was observed in either systolic, diastolic left ventricular function or LVMi following treatment with high-dose eplerenone for a duration of 26 weeks in a cohort of high-risk type 2 diabetes patients without known heart failure.

The increased risk of cardiovascular disease following the diagnosis of type 2 diabetes is well-established, with the concomitant diagnose of heart failure further substantially impairing prognosis [26]. The clinical implication of the improvement of diastolic dysfunction has been underlined by a recent report from the PARAGON-trial, elucidating the HFpEF-specific cardiovascular characteristics prior to developing HFrEF. Although the effects of the combination of sacubitril/neprilysin failed to achieve statistical significance on the composite endpoint of cardiovascular death and total heart failure hospitalizations [27], increased baseline-levels of LVMi, E/e’ ratio, atrial size, and pulmonary arterial systolic pressure were found as the greatest predictors of a subsequent cardiovascular event [28]. In the present trial, however, no observable benefit was found after high-dose MRA treatment using established measures of diastolic cardiac function: E/e’-ratio, E/A-ratio and left atrial volume. The absence of an observable effect of an MRA on diastolic function, contrasts previous findings showing an improvement in diastolic function among patients with metabolic syndrome and among patient with early signs of diabetic cardiomyopathy [29, 30]. A plausible explanation as to the absence of change is the relatively low proportion of patients with diastolic dysfunction in the current trial, with an abnormal E/e’ and increased atrial volume present in only 31% and 11%, respectively, which differs markedly from previous reports. Indeed, two meta-analyses summarizing the effects of MRAs on diastolic function in patients with HFpEF [15] and HFrEF [16], respectively, show the greatest effects are obtained in cohorts with diastolic dysfunction at enrollment [31], and further showing changes in E/e’, E/A and left atrial volume as the most consistent results following MRA treatment [15, 16]. Although the MIRAD trial used a NT-proBNP cut-of previously validated in type 2 diabetes [23], the cut-of is lower than what is currently used in HFpEF and HFrEF trials [32], and the resultant low proportion of diastolic dysfunction may reduce the study’s ability to detect a clinically relevant difference given the size and duration of the trial despite using high-dose therapy.

We did not observe an effect of high-dose MRA therapy on systolic ventricular function in this cohort without HFrEF. The lack of a change in a cohort of patients in stable antidiabetic and antihypertensive treatment without overt systolic dysfunction at baseline is in line with previous publications, where trials have yet to observe a consistent improvement in LVEF in patients without HFrEF following MRA therapy [15, 16]. Similarly, although a previous report has shown a beneficial effect of MRAs on GLS among patients with metabolic syndrome [29], no change was apparent in the current substudy. Even though an overall improvement in left ventricular function therefore was not evident in the present trial, the link between cardiovascular function and metabolic dysfunction remains of interest and may be key to unlocking a benefit prior to the development of HFrEF [33, 34]. Indeed, a recent publication from the HOMAGE trial, investigating increased doses of the MRA, spironolactone, in patients without heart failure, of whom 42% had diabetes, reported improvements in diastolic function [35]. Further, substantiating these results, recent results from the FIDELIO and FIGARO trials [36, 37], investigating the third-generation, dihydropyridine-based MRA, finerenone, have provided new evidence underlining the clinical benefit of MRA therapy in patient with type 2 diabetes and persistent albuminuria. The pooled-analysis of the trials, FIDELITY [38], showed that treatment caused a 14% and 23% decrease in the composite cardiovascular and renal clinical outcome, respectively, with the cardiovascular composite largely driven by a decrease in hospitalization for heart failure. As a consequence, finerenone is currently recommended as add-on to RAS-blockade in case of albuminuria [39].

Although MRAs have long been used as antihypertensives, experimental studies in animal models investigating the underlying mechanisms of MRA therapy have demonstrated extra-renal, receptor-mediated effects shown to be independent of the expected diuretic and antihypertensive effects mediated by mineralocorticoid receptor activation in the distal tubules of the kidneys [8–10]. Furthermore, an exploratory analysis from the HOMAGE trial using targeted proteomics has found evidence of the spironolactone exerting pleiotropic effects across multiple mechanistic pathways in humans, spanning the mitigation of fibrosis, thrombosis, fluid congestion, vascular function, and inflammation [40]. In recent years, cardiac magnetic resonance imaging has increased the scientific focus on LVM regression as an independent therapeutic target as well as a modifiable risk factor [41], with recent reports of decreases observed following SGLT2-i therapy [42, 43]. With the expression of the mineralocorticoid receptor in cardiomyocytes and vascular endothelial cells, a direct modulatory effect is possible, however, a significant decrease in LVM was not evident in the current study when evaluated by echocardiography. Although, LVM regression has been demonstrated as an effect of high-dose MRA therapy using cardiac magnetic resonance imaging in a subset of the patients in the current cohort [44], and findings are further corroborated by reports from a selective cohort of hypertensive patients using a similar design using magnetic resonance imaging [20], the current study’s inability to corroborate findings across multiple modalities may weaken the clinical implication of the small-to-moderate decrease in LVM seen with MRA treatment [44]. Similarly, MRA therapy has been proposed as attenuating right ventricular dysfunction and mitigating the effects in the early stages of pulmonary arterial hypertension [45], and a significant decrease in right ventricular GLS was observed in the current study as an effect of treatment. GLS in general has previously been shown to provide incremental prognostic value in patients with type 2 diabetes [24, 46], and may therefore capture additional markers of risk not easily detected using a conventional echocardiography protocol. However, the available measures of right ventricular function did not corroborate a benefit specific to right ventricular function nor was a change in tricuspid regurgitation velocity detectable in the current study. Although we did not measure mean arterial pulmonary pressure, which could further elucidate on conditions specific to the right ventricule, the overall clinical implication of a solitary change in a marker of right ventricular function is uncertain and in need of further study.

The clinical benefit of finerenone as shown by the pooled-analysis, FIDELITY, as well as the recent advances in outlining the mechanism of extra-renal MRA therapy [40] has taken great strides necessary to extend the benefits of MRA therapy beyond the HFrEF-phenotype. However, further study is needed to substantiate the clinical potential of MRA therapy. Consequently, the ongoing SPIRRIT trial have set out to re-investigate the role of spironolactone in HFpEF (ClinicalTrial.gov: NCT02901184), including patients with symptoms of heart failure and LVEF above 40%. Furthermore, the recently initiated FINEARTS-HF (ClinicalTrial.gov: NCT04435626), investigating the width of the HFpEF phenotype, is powered to detect changes in incidence of first and recurrent hospitalization due to heart failure and cardiovascular mortality, and results are expected to highlight the potential of MRAs as preventive of HFrEF and possibly as a therapeutic in HFpEF.

### Limitations

The present study is a small, single-center study with a duration of only 6 months, and thus, is restricted in its ability to predict long-term changes in risk. Furthermore, the study is an exploratory analysis based on data from the MIRAD trial. Although the study focuses on left ventricular diastolic dysfunction, all outcomes are either secondary outcomes prespecified as part of trial design or defined post-hoc. As an effect – and in consideration of multiplicity of testing—results must be viewed as hypothesis-generating and in need of corroboration. Finally, the present study contained a large proportion of patients with no signs of either overt systolic or diastolic function, with a long-standing stable clinical condition, which lowers the expected effects size of treatment. Considering the results of PARAGON trial [28], a cohort based on inclusion-criteria, which were highlighted as the greatest predictors of cardiovascular events in patients with HFpEF (e.g., higher LVMi, E/e’ ratio, left atrium size or pulmonary arterial systolic pressure) may provide a better framework to detect the absolute changes in response to treatment in study designs of similar size and duration in the future.

## Conclusion

In patients with type 2 diabetes without HFrEF, the addition of high-dose eplerenone to standard therapy for a duration of 26 weeks was not associated with a detectable improvement in left ventricular systolic or diastolic function when measured using echocardiography.

## Supplementary Information

**Additional file 1.**

## Data Availability

The datasets generated and/or analysed during the current study are not publicly available due to national restrictions in sharing patient-sensitive material, but are available from the corresponding author on reasonable request.

## Abbreviations

MRA: Mineralocorticoid Receptor Antagonist
LVMi: Left Ventricular Mass indexed to body surface area
GLS: Global Longitudinal Strain
LVEF: Left Ventricular Ejection Fraction
HFpEF: Heart Failure with Preserved Ejection Fraction
HFrEF: Heart Failure with Reduced Ejection Fraction
MIRAD: MIneralocorticoid Receptor Antagonist in type 2 Diabetes
EDV: End Diastolic Volume
ESV: End-Systolic Volume
SV: Stroke Volume
