# Supplementary material for: Effect on cardiac function among patients with type 2 diabetes following high-dose mineralocorticoid receptor antagonist using echocardiography; data from the MIRAD randomized clinical trial
Source: BMC Cardiovasc Disord. 2023 Mar 31;23:175. doi: 10.1186/s12872-023-03183-1 (PMC10064675; doi:10.1186/s12872-023-03183-1)
Supplement: Supplementary file 1 — Additional file 1. [file 12872_2023_3183_MOESM1_ESM.docx]

**
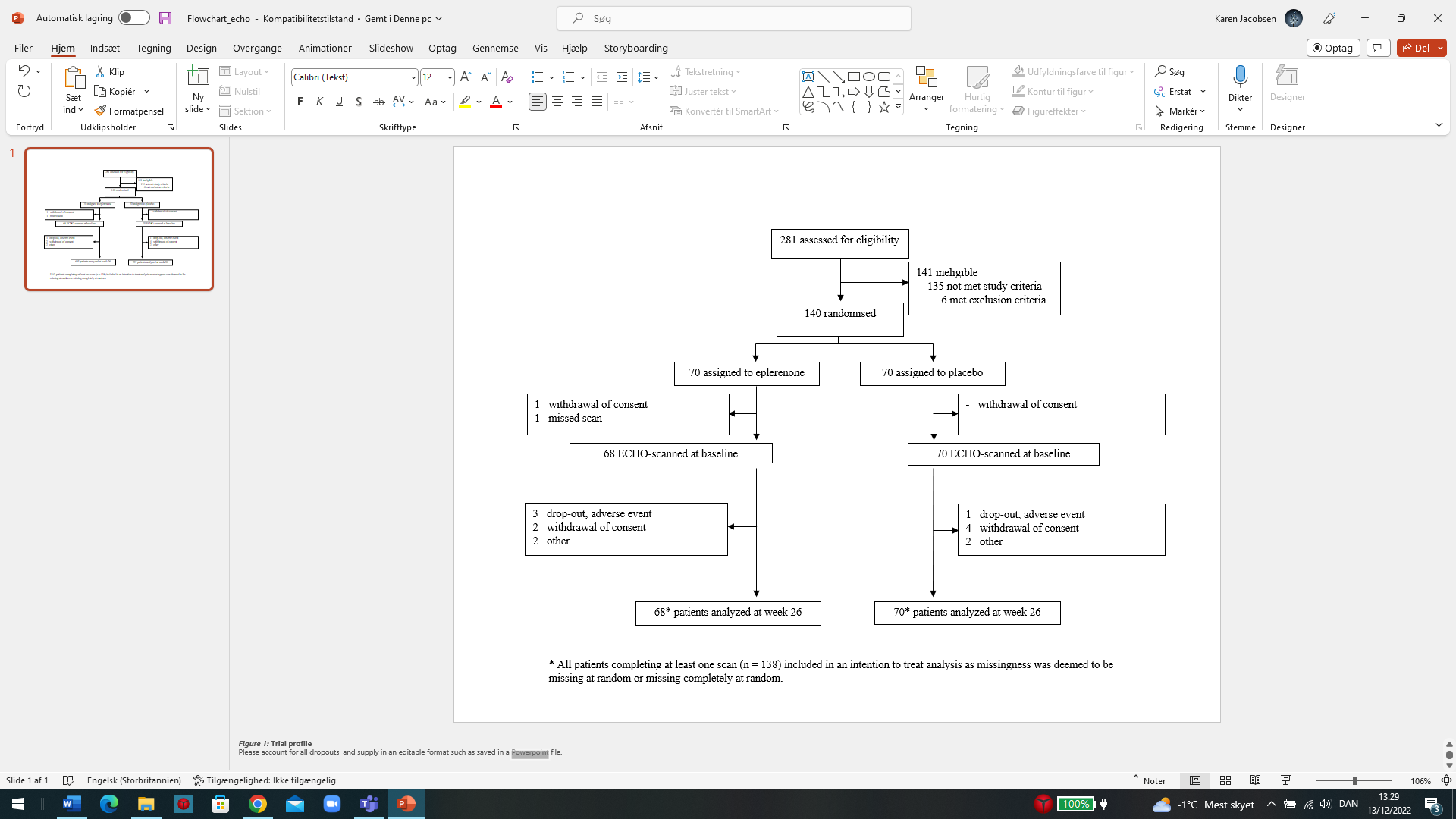
Supplementary Figure 1: Flowchart of patient participation in the trial**

**Supplementary table 1: Additional echocardiographic measures at baseline**

|  | Placebo | | Eplerenone | |
| --- | --- | --- | --- | --- |
| Structure |  |  |  |  |
| IVSD, cm | 1.2 | (0.2) | 1.2 | (0.2) |
| LVID, cm | 4.4 | (0.6) | 4.4 | (0.6) |
| LVPW, cm | 1.2 | (0.2) | 1.2 | (0.2) |
| Left ventricular function |  |  |  |  |
| *Systolic function* |  |  |  |  |
| EDV, mL | 84.2 | (22.6) | 90.7 | (23.0) |
| ESV, mL | 35.7 | (13.9) | 37.5 | (12.8) |
| Heart rate, beat/min | 72.0 | (13.3) | 69.5 | (11.5) |
| *Diastolic function* |  |  |  |  |
| e', cm/s | 6.9 | (1.7) | 7.4 | (1.7) |
| E-wave, m/s | 0.7 | (0.2) | 0.8 | (0.2) |
| A-wave, m/s | 0.8 | (0.2) | 0.8 | (0.2) |
| E/A-ratio | 1.1 | (0.6) | 1.1 | (0.4) |
| E-wave deceleration time, ms | 260.9 | (73.9) | 268.3 | (72.1) |
| Right ventricular function |  |  |  |  |
| GLS, % | -16.1 | (5.3) | -16.6 | (5.6) |
| S', cm/s | 12.2 | (3.0) | 12.0 | (2.6) |
| TAPSE, mm/s | 22.6 | (3.8) | 22.5 | (4.8) |

Additional echocardiographic values measured at baseline. Abbreviations: IVSD, intraventricular septal diameter at end-diastole; LVID, left ventricular internal diameter at end-diastole; LVPW, left ventricular posterior wall diameter at end-diastole; EDV end-diastolic volume; ESV, end-systolic volume; GLS, global longitudinal strain; TAPSE, triscupid annular plane systolic excursion

**Supplementary table 2: Analyses of conventional echocardiographic measures of cardiac function**

|  | Baseline | |  | Placebo | |  | P | Eplerenone | |  | P | Treatment effect | | | | P | |
| --- | --- | --- | --- | --- | --- | --- | --- | --- | --- | --- | --- | --- | --- | --- | --- | --- | --- |
| **Structure** |  |  |  |  |  |  |  |  |  |  |  |  |  |  |  | |  |
| IVSD, cm | 1.2 | [1.2 to | 1.2] | 0.0 | [0.0 to | 0.1] | 0.370 | 0.0 | [-0.1 to | 0.0] | 0.922 | 0.0 | [-0.1 to | 0.0] | 0.445 | |  |
| LVID, cm | 4.4 | [4.3 to | 4.5] | 0.0 | [-0.1 to | 0.2] | 0.543 | 0.0 | [-0.1 to | 0.1] | 0.887 | 0.0 | [-0.2 to | 0.1] | 0.728 | |  |
| LVPW, cm | 1.2 | [1.2 to | 1.2] | 0.0 | [-0.1 to | 0.0] | 0.586 | 0.0 | [-0.1 to | 0.0] | 0.135 | 0.0 | [-0.1 to | 0.0] | 0.461 | |  |
| **Systolic function** |  |  |  |  |  |  |  |  |  |  |  |  |  |  |  | |  |
| LV SV, mL | 50.8 | [48.6 to | 53.1] | 1.5 | [-1.3 to | 4.2] | 0.287 | 0.1 | [-2.7 to | 2.8] | 0.971 | -1.4 | [-5.1 to | 2.3] | 0.445 | |  |
| **Diastolic function** |  |  |  |  |  |  |  |  |  |  |  |  |  |  |  | |  |
| A’, cm/s | 9.6 | [9.2 to | 10.0] | -0.3 | [-0.8 to | 0.2] | 0.218 | 0.2 | [-0.3 to | 0.7] | 0.444 | 0.5 | [-0.2 to | 1.1] | 0.140 | |  |
| S’, cm/s | 7.1 | [6.9 to | 7.4] | 0.0 | [-0.3 to | 0.4] | 0.790 | 0.2 | [-0.1 to | 0.5] | 0.188 | 0.2 | [-0.3 to | 0.6] | 0.427 | |  |
| **Right ventricular function** |  |  |  |  |  |  |  |  |  |  |  |  |  |  |  | |  |
| GLS, %* | -16.6 | [-17.6 to | -15.6] | 1.4 | [0.0 to | 2.8] | 0.057 | -1.2 | [-2.7 to | 0.3] | 0.107 | -2.6 | [-4.5 to | -0.7] | 0.009 | |  |
| S’, cm/s | 12.1 | [11.6 to | 12.6] | -0.1 | [-0.8 to | 0.6] | 0.790 | 0.0 | [-0.7 to | 0.8] | 0.916 | 0.1 | [-0.8 to | 1.1] | 0.774 | |  |
| TAPSE, mm/s | 22.6 | [21.9 to | 23.4] | -0.4 | [-1.6 to | 0.8] | 0.482 | 0.3 | [-0.9 to | 1.5] | 0.636 | 0.7 | [-0.9 to | 2.3] | 0.378 | |  |

Treatment effects across additional measures of cardiovascular function measured at baseline and after 26 weeks. The analyses are performed using a constrained linear mixed model*.* Abbreviations: IVSD, intraventricular septal diameter at end-diastole; LVID, left ventricular internal diameter at end-diastole; LVPW, left ventricular posterior wall at end-diastole; LV SV, left ventricular stroke volume; GLS, global longitudinal strain; TAPSE, tricuspid annular plane systolic excursion.

**Supplementary table 3: Complete case analyses of effect of high-dose eplerenone after 26 weeks of treatment**

|  | Baseline | |  | Placebo | |  | P | Eplerenone | |  | P | Treatment effect | | | | P | |
| --- | --- | --- | --- | --- | --- | --- | --- | --- | --- | --- | --- | --- | --- | --- | --- | --- | --- |
| Structure |  |  |  |  |  |  |  |  |  |  |  |  |  |  |  | |  |
| LVMi. g/m2 | 92.4 | [88.4 to | 96.4] | 2.0 | [-2.8 to | 6.8] | 0.401 | -1.7 | [-6.6 to | 3.1] | 0.481 | -3.8 | [-10.2 to | 2.7] | 0.247 | |  |
| IVS, cm | 1.2 | [1.2 to | 1.3] | 0.0 | [0.0 to | 0.1] | 0.459 | 0.0 | [-0.1 to | 0.0] | 0.811 | 0.0 | [-0.1 to | 0.0] | 0.444 | |  |
| LVID, cm | 4.4 | [4.3 to | 4.5] | 0.0 | [-0.1 to | 0.2] | 0.461 | 0.0 | [-0.1 to | 0.2] | 0.784 | 0.0 | [-0.2 to | 0.1] | 0.728 | |  |
| LVPW, cm | 1.2 | [1.2 to | 1.2] | 0.0 | [-0.1 to | 0.0] | 0.549 | 0.0 | [-0.1 to | 0.0] | 0.124 | 0.0 | [-0.1 to | 0.0] | 0.461 | |  |
| Left ventricular function |  |  |  |  |  |  |  |  |  |  |  |  |  |  |  | |  |
| GLS, %* | -15.1 | [-15.7 to | -14.5] | 0.5 | [-0.3 to | 1.3] | 0.240 | 0.0 | [-0.7 to | 0.8] | 0.905 | -0.4 | [-1.5 to | 0.6] | 0.427 | |  |
| EF, %* | 59.0 | [57.7 to | 60.4] | 0.0 | [-1.6 to | 1.6] | 0.972 | 0.9 | [-0.7 to | 2.5] | 0.273 | 0.9 | [-1.1 to | 2.8] | 0.382 | |  |
| EDV, mL | 87.4 | [83.2 to | 91.6] | 1.9 | [-2.3 to | 6.1] | 0.376 | -2.1 | [-6.3 to | 2.1] | 0.328 | -3.9 | [-9.6 to | 1.7] | 0.167 | |  |
| ESV, mL | 36.3 | [34.0 to | 38.5] | 0.2 | [-2.1 to | 2.6] | 0.837 | -1.7 | [-4.1 to | 0.6] | 0.145 | -2.0 | [-5.0 to | 1.1] | 0.201 | |  |
| SV, mL | 51.1 | [48.7 to | 53.6] | 1.4 | [-1.4 to | 4.2] | 0.330 | -0.1 | [-2.8 to | 2.7] | 0.965 | -1.4 | [-5.1 to | 2.3] | 0.445 | |  |
| Heart rate, beat/min | 70.3 | [68.0 to | 72.6] | 1.3 | [-1.1 to | 3.7] | 0.296 | 0.5 | [-2.0 to | 3.0] | 0.671 | -0.7 | [-3.9 to | 2.4] | 0.643 | |  |
| Diastolic function |  |  |  |  |  |  |  |  |  |  |  |  |  |  |  | |  |
| E-wave, m/s | 0.8 | [0.7 to | 0.8] | 0.0 | [0.0 to | 0.0] | 0.563 | 0.0 | [0.0 to | 0.0] | 0.897 | 0.0 | [0.0 to | 0.1] | 0.749 | |  |
| A-wave, m/s | 0.8 | [0.7 to | 0.8] | 0.0 | [0.0 to | 0.0] | 0.484 | 0.0 | [0.0 to | 0.1] | 0.158 | 0.0 | [0.0 to | 0.1] | 0.129 | |  |
| E/A-ratio | 1.1 | [1.0 to | 1.2] | 0.0 | [-0.1 to | 0.1] | 0.719 | -0.1 | [-0.1 to | 0.0] | 0.152 | -0.1 | [-0.2 to | 0.0] | 0.193 | |  |
| e', cm/s | 7.1 | [6.8 to | 7.4] | 0.0 | [-0.3 to | 0.4] | 0.893 | 0.1 | [-0.2 to | 0.5] | 0.483 | 0.1 | [-0.4 to | 0.6] | 0.669 | |  |
| E/e’-ratio | 11.2 | [10.4 to | 12.0] | 0.0 | [-1.6 to | 1.6] | 0.972 | 0.9 | [-0.7 to | 2.5] | 0.273 | 0.0 | [-1.2 to | 1.2] | 0.991 | |  |
| E-wave desceleration time | 266.0 | [252.5 to | 279.4] | 1.7 | [-15.1 to | 18.5] | 0.844 | 13.3 | [-3.6 to | 30.3] | 0.122 | 11.7 | [-9.7 to | 33.1] | 0.283 | |  |
| A’, cm/s | 9.6 | [9.2 to | 10.0] | -0.3 | [-0.8 to | 0.2] | 0.198 | 0.2 | [-0.3 to | 0.6] | 0.481 | 0.5 | [-0.2 to | 1.1] | 0.140 | |  |
| S’, cm/s | 7.1 | [6.8 to | 7.4] | 0.0 | [-0.3 to | 0.4] | 0.760 | 0.2 | [-0.1 to | 0.5] | 0.178 | 0.2 | [-0.3 to | 0.6] | 0.427 | |  |
| LA volume. mL | 53.1 | [49.7 to | 56.4] | -2.1 | [-5.2 to | 1.1] | 0.195 | -4.4 | [-7.6 to | -1.3] | 0.006 | -2.3 | [-6.6 to | 1.9] | 0.279 | |  |
| Right ventricular function |  |  |  |  |  |  |  |  |  |  |  |  |  |  |  | |  |
| GLS, %* | -16.5 | [-17.6 to | -15.4] | 1.4 | [0.0 to | 2.9] | 0.048 | -1.1 | [-2.6 to | 0.3] | 0.130 | -2.6 | [-4.5 to | -0.7] | 0.008 | |  |
| S’, cm/s | 12.0 | [11.5 to | 12.6] | -0.1 | [-0.8 to | 0.6] | 0.845 | 0.1 | [-0.7 to | 0.8] | 0.861 | 0.1 | [-0.8 to | 1.1] | 0.771 | |  |
| TAPSE, mm/s | 22.5 | [21.7 to | 23.4] | -0.4 | [-1.6 to | 0.8] | 0.537 | 0.3 | [-0.9 to | 1.5] | 0.583 | 0.7 | [-0.9 to | 2.3] | 0.379 | |  |
| Complete case analysis regarding effects of treatment on all reported endpoints. The analyses are performed using a constrained linear mixed model. Abbreviations: LVMi, left ventricular mass indexed to body surface area; IVSD, intraventricular septal diameter at end-diastole; LVID, left ventricular internal diameter at end-diastole; LVPW, left ventricular posterior wall diameter at end-diastole; GLS, global longitudinal strain; EF, ejection fraction; EDV, end-diastolic volume; ESV, end-systolic volume; SV, stroke volume; LA, left atrial volume; TAPSE, tricuspid annular plane systolic excursion. | | | | | | | | | | | | | | | | |  |

**Supplementary table 4: Testing for effect medication of prescribed medication on diastolic function and left ventricular mass**

|  | **Treatment** | **Prescribed** |  |  | **P-value** | **Not prescribed** |  |  | **P-value** | **Interaction** |
| --- | --- | --- | --- | --- | --- | --- | --- | --- | --- | --- |
| LAi, mL/m2 | ACE-i | 1.4 | [-2.5 to 5.2] | | 0.485 | -2.0 | [-4.4 to 0.3] | | 0.094 | 0.142 |
|  | ARB | -2.3 | [-5.1 to 0.5] | | 0.111 | 0.1 | [-2.8 to 2.9] | | 0.953 | 0.248 |
|  | Beta-blockade | -3.1 | [-6.4 to 0.2] | | 0.064 | 0.1 | [-2.5 to 2.6] | | 0.965 | 0.132 |
| E/e', % | ACE-i | 1.0 | [-1.3 to 3.2] | | 0.385 | -0.4 | [-1.8 to 1.0] | | 0.561 | 0.296 |
|  | ARB | 0.1 | [-1.6 to 1.8] | | 0.900 | -0.1 | [-1.8 to 1.6] | | 0.889 | 0.851 |
|  | Beta-blockade | 0.6 | [-1.3 to 2.6] | | 0.521 | -0.4 | [-1.9 to 1.1] | | 0.620 | 0.418 |
| LVMi, g/m2 | ACE-i | -6.0 | [-18.1 to 6.2] | | 0.332 | -2.9 | [-10.6 to 4.8] | | 0.455 | 0.676 |
|  | ARB | -5.7 | [-14.9 to 3.5] | | 0.221 | -1.7 | [-10.9 to 7.5] | | 0.713 | 0.541 |
|  | Beta-blockade | -1.8 | [-12.6 to 9.0] | | 0.742 | -4.6 | [-12.6 to 3.4] | | 0.254 | 0.677 |

Treatment effects of the primary focus are tested for the presence of effect modification of concurrent therapy prescribed at baseline, first according to the presence or absence and finally a formal test of interaction. The analyses are performed using a modified constrained linear mixed model expanded the primary analysis to include the fixed effects: ‘*time-precribed interaction’* and ‘*time-prescribed-treatment interaction’.* Abbreviations: LAi, left atrial volume indexed to body surface area; LVMi, left ventricular mass indexed to body surface area; ACE-i, angiotensin converting enzyme; ARB, angiotensin receptor blocker.
